# Interpretable Machine Learning Models for Analyzing Determinants Affecting the Use of mHealth Apps Among Family Caregivers of Patients With Stroke in Chinese Communities: Cross-Sectional Survey Study

**DOI:** 10.2196/73903

**Published:** 2025-11-24

**Authors:** Yun Du, Jun-Ying Fan, Guang-Zhi Liu, Zi-Yue Yang, Yang Lei, Yu-Fang Guo

**Affiliations:** 1 School of Nursing and Rehabilitation, Shandong University, 44 Wen Hua Xi Road, Jinan, Shandong, 250012, China, 86 15269163352; 2 Department of Neurology, The Affiliated Hospital of Xuzhou Medical University, Xuzhou, China; 3 School of Nursing, Nanjing Medical University, Nanjing, China

**Keywords:** caregivers, mHealth, community, influencing factors, machine learning model, stroke, mobile health

## Abstract

**Background:**

Mobile health (mHealth) apps are believed to be an effective method to support family caregivers to better care for patients with stroke. This study’s purpose was to explore the status and the influencing factors of mHealth app use among family caregivers of patients with stroke via machine learning (ML) models.

**Objective:**

This study aimed to understand the status quo of mHealth app use among community family caregivers of patients with stroke and the factors influencing their use behavior. Six ML models were used to construct the classifier, and the Shapley Additive Explanations (SHAP) algorithm was introduced to interpret the best ML model.

**Methods:**

In this cross-sectional study, family carers of patients with stroke were recruited. Data on their basic profile and mHealth app use were obtained through face-to-face questionnaires. Hedonic motivation, usage habits, and other relevant information were additionally measured among app users. A total of 12 models were constructed using six ML algorithms. The top-performing logistic regression and random forest models were further analyzed with SHAP to interpret key influencing factors.

**Results:**

A total of 360 family caregivers of patients with stroke were included in this study from March 2023 to November 2023, of which 206 (57.2%) reported having used mHealth apps. Of the 6 ML models, the logistic regression model performed the best in terms of whether caregivers used the mHealth app, with an area under the receiver operating characteristic curve of 0.753 (95% CI 0.698‐0.802), accuracy of 0.694 (95% CI 0.647‐0.742), sensitivity of 0.748 (95% CI 0.688‐0.806), and specificity of 0.623 (95% CI 0.547‐0.698). SHAP analysis showed that the top 5 most influencing factors were educational level, age, the patient’s self-care ability, the relationship with the cared-for individual, and the duration of illness. The random forest model performed best in terms of use behavior with an area under the receiver operating characteristic curve of 0.773 (95% CI 0.725‐0.818), accuracy of 0.602 (95% CI 0.534‐0.665), sensitivity of 0.476 (95% CI 0.420‐0.533), and specificity of 0.769 (95% CI 0.738‐0.797). The SHAP analysis revealed that hedonic motivation, habits, occupation, convenience conditions, and effort expectations were the 5 most significant influencing factors.

**Conclusions:**

The research results indicate that the software developers and policymakers of mHealth apps should take the abovementioned influencing factors into consideration when developing and promoting the software. We should focus on the older adults with lower educational levels, lower the threshold for software use, and provide more convenient conditions. By grasping the hedonistic tendencies and habitual usage characteristics of users, they can provide them with more concise and accurate health information, which will enhance the popularity and effectiveness of mHealth apps.

## Introduction

Stroke is a central nervous system disorder characterized by acute vascular damage, which is a leading cause of disability and death globally [1,2]. In recent years, despite advancements in medical care that have contributed to reduced stroke mortality rates, more than 75% of surviving patients with stroke still have moderate or severe functional disabilities in limbs, cognition, and language [3-5], which result in patients having limited ability to perform daily life activities and needing prolonged rehabilitation and treatment. Nevertheless, owing to economic and medical limitations, over 80% of patients with stroke opt for home rehabilitation once their condition stabilizes [6], with care predominantly provided by family members [7]. Notably, 25% of stroke survivors were readmitted to the hospital within 90 days, and 73% of patients with stroke experienced falls within 6 months, with 4% sustaining fractures during these incidents, attributed to improper care practices or lack of professional guidance in caregiving [8,9]. Family caregivers of patients with stroke experienced a deficiency in disease knowledge, inadequate caregiving skills, heightened caregiving stress, poor role adjustment, and insufficient social support [10-12]. This adversely impacted the rehabilitation process, diminished patients’ quality of life, and escalated the burden on caregivers [13]. Consequently, assisting family caregivers of patients with stroke in obtaining knowledge about the condition and efficient management strategies has emerged as a pressing issue.

The widespread adoption of smartphones and advancements in digital health technology present significant possibilities for the management of stroke through telemedicine and mobile health (mHealth) technologies [14,15]. mHealth apps, a primary manifestation of mHealth, are apps for mobile phones or tablets designed for health care research and services aimed at enhancing patient health outcomes [16]. mHealth apps offer users affordable, high-quality, round-the-clock access to evidence-based health information worldwide and improve patient disease management through behavioral change models [17-19]. Numerous studies supported that mHealth apps used by family caregivers of patients with stroke for disease management enhanced caregivers’ understanding of the condition, refined their caregiving skills and efficiency, and positively influenced their psychological well-being, thereby benefiting the patients’ recovery process [20,21].

However, most of the studies focused on the validation of the effects of mHealth app use by family carers of patients with stroke, and the study design was also mostly intervention-based, with limitations in external validity. It is important to recognize that utilization thresholds exist for the use of mHealth apps. Disparities in the quantity and quality of knowledge obtained among users during their use are evident [22]. Multiple contributory factors interact with discrepancy, including personal, familial, economic, and other real-world elements [23]. These factors interact to ascertain that the mHealth app can deliver its full potential and genuinely benefit patients and users. However, there is still a lack of clarity regarding the real-world use of mHealth apps by family caregivers of patients with stroke in nonintervention states and the factors influencing whether they use them or not.

In this study, machine learning (ML) algorithms were used to explore the factors influencing their use and use behavior, and the ML approach demonstrated significant advantages over traditional linear regression models. These algorithms can capture more complex nonlinear relationships and have stronger feature selection capabilities and better predictive performance [24]. Therefore, the choice of ML algorithms enables a more comprehensive exploration of influencing factors. However, there is still the disadvantage of limited model interpretability, so this study applied the Shapley Additive Explanations (SHAP) algorithm based on ML algorithms to enhance model interpretability and facilitate a better understanding of the model and results.

In summary, this study applied ML algorithms to investigate the current status of mHealth app use among family caregivers of patients with stroke in the community and the factors influencing usage behavior. The objectives were to provide empirical data to support health care providers and technology developers in focusing on populations who underuse mHealth apps. By gaining a deeper understanding of the real-world barriers these groups face, we expected to be able to provide them with more targeted assistance to increase their usage of mHealth apps and optimize use patterns. This will help ensure that mHealth apps reach a wider audience of patients with stroke and their family caregivers, improving the effectiveness of disease management.

## Methods

### Study Design and Participants

This study was a cross-sectional survey. The convenience sampling method was used to select family carers of patients with stroke who met the inclusion criteria for the study. A total of 360 eligible family carers of patients with stroke were included.

The following patient inclusion criteria were used: (1) patients with stroke who met the diagnostic criteria of stroke [25] and were diagnosed by cranial computed tomography or magnetic resonance imaging, (2) aged ≥18 years, (3) conscious and with the ability to use words or clearly express their wishes, and (4) informed consent and voluntary participation in the survey. Those who are too ill to cooperate with the investigation are excluded.

Inclusion criteria for family caregivers were as follows:

1. Primary caregiver of the patient with stroke: the person who is related to the patient and who is responsible for the patient’s primary care (the person who cares for the patient for the longest time during the day). If there are several primary caregivers at the same time, the patient will designate one of them as the primary caregiver.
2. Aged ≥18 years
3. Informed consent and voluntary participation in the survey

Paid professional caregivers were excluded.

### Sample Size Calculation

This study adopted the sample size calculation method based on the 10-fold events per variable (EPV) principle [26,27]. When constructing the model regarding the use of mHealth apps, a total of 10 variables were included. According to the survey results, the use rate of mHealth apps was 57.22%. According to the EPV principle, at least 175 samples were required. When constructing the model for the use behavior of mHealth apps, a total of 8 variables were included. Based on the clustering results, the expected occurrence rate (cluster 3) was 51.5% (Figure S2 in Multimedia Appendix 1). According to the EPV principle, at least 156 samples were needed. This study ultimately included 360 family caregivers of patients with stroke, among whom 206 had used mHealth apps.

### Data Collection

Data collection for this study was conducted between March 2023 and November 2023 in 4 community health centers in Jinan, Shandong Province and Xuzhou, Jiangsu Province. Patients with stroke who had ever been diagnosed by neurological specialists were selected among the population attending the community hospitals, and their primary caregivers who met the criteria were included in the study after reviewing the electronic records, resulting in the inclusion of 360 study participants. For those who used mHealth apps, after completing the basic questionnaire, a further questionnaire was completed to investigate their use behaviors. Data were collected face-to-face by a uniformly trained research team using a standardized paper questionnaire, during which the questionnaire entries were interpreted item by item, respondents’ questions were uniformly explained, and answers were recorded after verbal confirmation was obtained. The completeness of each questionnaire was verified immediately after completion, and omissions were found to be added on the spot, resulting in 360 questionnaires being recovered and zero invalid questionnaires being eliminated, giving an effective recovery rate of 100%. This quality control process ensures the integrity and reliability of the data.

### Influencing Factors

The influencing factors included general sociodemographic data and disease-related data, where the disease-related data were divided into 2 parts: the patient part included the type of stroke (ischemic and hemorrhagic), whether or not they were taking medication, whether or not they had adjusted their lifestyle after the illness, whether or not they were undergoing rehabilitation, whether or not they had been regularly reexamined, the number of sequelae of the stroke, the number of diseases other than stroke, the number of years of the patient’s life with a diagnosis of stroke, the number of years since the stroke, and the patient’s ability to care for themselves as measured by the Barthel Index [28]. The carer section investigated the number of illnesses the carer had themselves. In addition to this, the type of mHealth app used was captured using the multiple-choice question “Which mHealth App or Apps do you usually use?” In continuing to explore the influences on users’ use behavior, standardized maturity scales measuring indicators of performance expectations, effort expectations, habituation, value, and hedonic motivation were included (Multimedia Appendix 1).

### Outcomes

Use of mHealth apps was measured using the question “Have you ever used mHealth software?” with responses specified as a dichotomous variable: “yes” and “no.” In addition to this, the frequency, duration, and familiarity of carers’ use of mHealth apps were measured using questions, which were used to represent the study participants’ use behavior in a combined manner.

### Statistical Analysis 

Python (version 3.11.3; Python Software Foundation) was used. Data were described and analyzed statistically, with categorical variables represented as frequencies and percentages.

The k-modes clustering algorithm was used to categorize the study participants using an mHealth app. Detailed information on the clustering algorithm can be found in Multimedia Appendix 1. The variables entering k-modes consensus clustering included frequency of use, length of use, and familiarity of use. Through multiple iterations, the data points were grouped into clusters with similar characteristics.

Subsequently, we used the elbow method to determine the optimal number of clusters (Multimedia Appendix 1). After determining the optimal clusters, study participants using mHealth apps were divided into groups with different use behaviors according to the clustering results.

ML algorithms were used to explore the factors influencing whether or not an mHealth app is used and its use behavior [29]. Six main classifiers were selected for testing in this study: logistic regression (LR), random forest (RF), support vector machine, extreme gradient boosting, light gradient boosting machine, and naive Bayes (Multimedia Appendix 1).

This study used the nested cross-validation method suitable for small-sample ML [30] (Multimedia Appendix 1). The outer loop is divided into 5 folds to evaluate performance, and the inner loop (divided into 5 layers) is used for hyperparameter tuning. To ensure the reliability of the evaluation results, reduce the randomness of the model, and improve the generalization ability of the model, different strategies are selected for feature selection based on different models in this study (Table S1 in Multimedia Appendix 1). The model’s overall performance was assessed using 4 metrics: accuracy, area under the receiver operating characteristic curve (AUROC), sensitivity, and specificity. The Brier score and calibration curve were reported to evaluate the reliability of the predicted probabilities. For each indicator, we combined the prediction results from all the outer test sets to calculate the overall indicator. The 95% CI was calculated through 1000 bootstrap samplings.

For the models exhibiting optimal performance, we used the SHAP algorithm to elucidate the prediction outcomes of the ML models (Multimedia Appendix 1). Among them, the SHAP value represents the minimum contribution of the feature to the model. The higher the SHAP, the greater the influence of the feature on the model. Consequently, using this method facilitates the interpretation of prediction results for each observation sample within the dataset and enhances our comprehension of the ML model. We used the bootstrap method to evaluate the stability of the SHAP values. We conducted 100 bootstrap iterations and recalculated the SHAP values after each sampling, thereby quantifying the range of fluctuations in feature importance.

A learning curve was drawn to assess the impact of sample size on model performance and diagnose whether the model is underfitting or overfitting. Highly correlated and low-correlated features were removed to evaluate the model’s robustness to feature changes and verify the stability of the feature selection strategy.

### Ethical Considerations

This study was approved by the local ethics committee (2023-R-036). Throughout the study, we strictly adhered to the principle of informed consent, fully informed the study participants about the study, and respected the respondents’ right to independently choose to participate in or withdraw from the study at any time. After the investigation is completed, we will give small gifts (such as towels and soaps) to the participants as a token of our gratitude.

## Results

### Sample Characteristics

Table 1 presents the demographic and disease-related characteristics of the participants. The age of participants ranged from 23 to 85 years, with a mean age of 61.93 (SD 15.08) years. Among the 360 participants, 54.4% (n=196) were women, 63.6% (n=229) had attained junior high or high school education, 97.2% (n=350) were married, 95% (n=342) resided in urban areas, 58.9% (n=212) were retired, and 85.6% (n=308) had a monthly income below CN ¥6000 (US $836.52). Furthermore, 92.8% (n=334) of caregivers cared for patients with ischemic stroke, and 51.9% (n=187) of carers cared for mildly dependent patients.

**Table 1.** Characteristics among caregivers (N=360).

Characteristics	Total, n (%)	Use (n=206), n (%)	Nonuse (n=154), n (%)	Chi-square (*df*)	*P* value
Demographic characteristics					
Gender				0.1 (1)	.72
Man	164 (45.6)	96 (46.6)	68 (44.2)		
Woman	196 (54.4)	110 (53.4)	86 (55.8)		
Age (y)				45.9 (1)	<.001
≤60	139(38.6)	111 (53.9)	28 (18.2)		
>60	221(61.4)	95 (46.1)	126 (81.8)		
Degree of education				61.7 (4)	<.001
Primary and below	62 (17.2)	15 (7.3)	47 (30.5)		
Junior high school	100 (27.8)	64 (31.1)	65 (42.2)		
Senior high school (technical secondary school)	129 (35.8)	66 (32)	34 (22.1)		
Junior college	40 (11.1)	34 (16.5)	6 (3.9)		
Bachelor’s degree and higher	29 (8.1)	27 (13.1)	3 (1.3)		
Marital status				3.2 (1)	.07
Spinsterhood	10 (2.8)	9 (4.4)	1 (0.7)		
Married	350 (97.2)	197 (95.6)	153 (99.4)		
Family place of residence				0.2 (1)	.70
Cities and towns	342 (95)	197 (95.6)	145 (94.2)		
Village	18 (5)	9 (4.4)	9 (5.8)		
Occupational status				29.0 (3)	<.001
Unemployed	46 (12.8)	22 (10.7)	24 (15.6)		
On job	91 (25.3)	74 (35.9)	17 (11)		
Retired	212 (58.9)	105 (51)	107 (69.5)		
Others	11 (3.1)	5 (2.4)	6 (3.9)		
Monthly income (¥)^a^				11.0 (4)	.03
<2000	69 (19.2)	29 (14.1)	40 (25.9)		
2000‐3999	105 (29.2)	63 (30.6)	42 (27.3)		
4000‐5999	134 (37.2)	77 (37.4)	57 (37)		
6000‐7999	31 (8.6)	22(10.7)	9 (5.8)		
>8000	21 (5.8)	15 (7.3)	6 (3.9)		
Patient-caregiver relationship				42.0 (2)	<.001
Spouse	256 (71.1)	121 (58.7)	135 (87.7)		
Parent	98 (27.2)	83 (40.3)	15 (9.7)		
Child	6 (1.7)	2 (1)	4 (2.6)		
Disease characteristics					
Stroke type				0.4 (1)	.50
Hemorrhagic	26 (7.2)	17 (8.2)	9 (5.8)		
Ischemic	334 (92.8)	189 (91.8)	145 (94.2)		
Rehabilitation measures				0.6 (1)	.43
Yes	350 (97.2)	202 (98.1)	148 (96.1)		
No	10 (2.8)	4 (1.9)	6 (3.9)		
Take drug				3.2 (1)	.07
Yes	30 (8.3)	12 (5.8)	18 (11.7)		
No	330 (91.7)	194 (94.2)	136 (88.3)		
Adjust lifestyle				2.3 (1)	.13
Yes	72 (20)	35 (17)	37 (24)		
No	288 (80)	171 (83)	117 (76)		
Rehabilitation therapy				0.1 (1)	.79
Yes	346 (96.1)	197 (95.6)	149 (96.8)		
No	14 (3.9)	9 (4.4)	5 (3.2)		
Regular reexamination				0.1 (1)	.71
Yes	348 (96.7)	198 (96.2)	150 (97.4)		
No	12 (3.3)	8 (3.8)	4 (2.6)		
Number of sequelae				0.6 (2)	.74
≤1	295 (81.9)	171 (83)	124 (80.5)		
2	41 (11.4)	23 (11.2)	18 (11.7)		
≥3	24 (6.7)	12 (5.8)	12 (7.8)		
Number of complications				3.8 (2)	15
≤1	154 (42.8)	93 (45.1)	61 (39.6)		.
2	117 (32.5)	70 (34)	47 (30.5)		
≥3	89 (24.7)	43 (20.9)	46 (29.9)		
Number of illnesses in caregivers				6.3 (3)	.10
≤1	219 (60.8)	136 (66)	83 (53.9)		
2	61 (16.9)	28 (13.6)	33 (21.4)		
3	44 (12.2)	22 (10.7)	22 (14.3)		
≥4	36 (10)	20 (9.7)	16 (10.4)		
Years of illness				6.8 (2)	.03
<10	252 (70)	154 (74.8)	98 (63.6)		
Approximately 10-19	77 (21.4)	40 (19.4)	37 (24)		
≥20	31 (8.6)	12 (5.8)	19 (12.3)		
Patient self-care ability				6.8 (3)	.08
Complete self-care	122 (33.9)	81 (39.3)	41 (26.6)		
Mild dependence	187 (51.9)	100 (48.5)	87 (56.5)		
Moderate dependence	38 (10.6)	18 (8.7)	20 (13)		
Heavily depend on	13 (3.6)	7 (3.4)	6 (3.9)		

a The exchange rate between the Chinese Yuan and US dollar is US $1=CN ¥7.1726. This rate is based on the midpoint of the interbank foreign exchange market on February 25, 2025, as published by the People’s Bank of China.

### Use of mHealth Apps Among Participants

Among the 360 participants, 206 of them had used mHealth apps, with a use rate of 57.2%. Among the caregivers who had used mHealth apps, 82.5% (n=170) caregivers used sports and health apps for monitoring steps, sleep, and obtaining relevant exercise guidance; 51.9% (n=107) caregivers used hospital-related platforms, mainly for registering, enquiring about fees, and checking examination results; 3.9% (n=8) caregivers used medical and health service apps, including learning medical knowledge, buying medicine, and checking examination results; 1% (n=2) of the caregivers used e-Medicare apps, mainly for inquiring about medical insurance information; and 1% (n=2) of the caregivers used apps connected with exercise equipment, for checking and analyzing the use of the equipment and adjusting the parameters.

### Cluster Analysis and 3-Cluster Features

**Figure 1.**
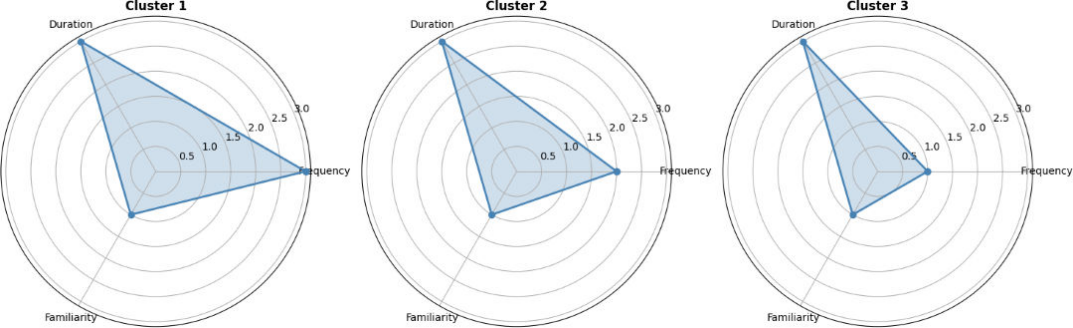
Radar map of the 3 clusters for characteristics.

The position of the elbow in the scree plot indicates the optimal number of clusters (k=3; Figure S1 in Multimedia Appendix 1). The silhouette coefficient is 0.608, the Calinski-Harabasz index is 127.679, and the ratio of intercluster/intracluster distance is 3.15. This indicates a reasonable clustering structure with significant differences between clusters. Therefore, the final value of k is determined to be 3. Furthermore, we used a radar map to represent these features, which is shown in Figure 1. There were no significant differences in the usage duration and familiarity between the 3 clusters. However, there were notable differences in the usage frequency. Cluster 1, which included 23.8% (48/203) of caregivers who used mHealth apps, had the lowest use frequency, while cluster 3, which included 51.5% (105/203) of caregivers who used mHealth apps, had the highest use frequency (Figure S2 in Multimedia Appendix 1).

### Factors Influencing Whether to Use mHealth Apps

The results of ML indicate that among the 6 models, LR demonstrates the best performance (Table S2 in Multimedia Appendix 1). Compared with other models, LR had the highest AUROC value (0.753), indicating that LR has the strongest comprehensive ability in classification decisions. Moreover, the accuracy, sensitivity, and specificity are all at a relatively high level, and they also balance sensitivity and specificity well. The calibration curve and the Brier score also suggest that LR is the best model (Figure S3 in Multimedia Appendix 1). Therefore, after considering all the indicators comprehensively, this study subsequently selects LR for interpretability analysis.

**Figure 2.**
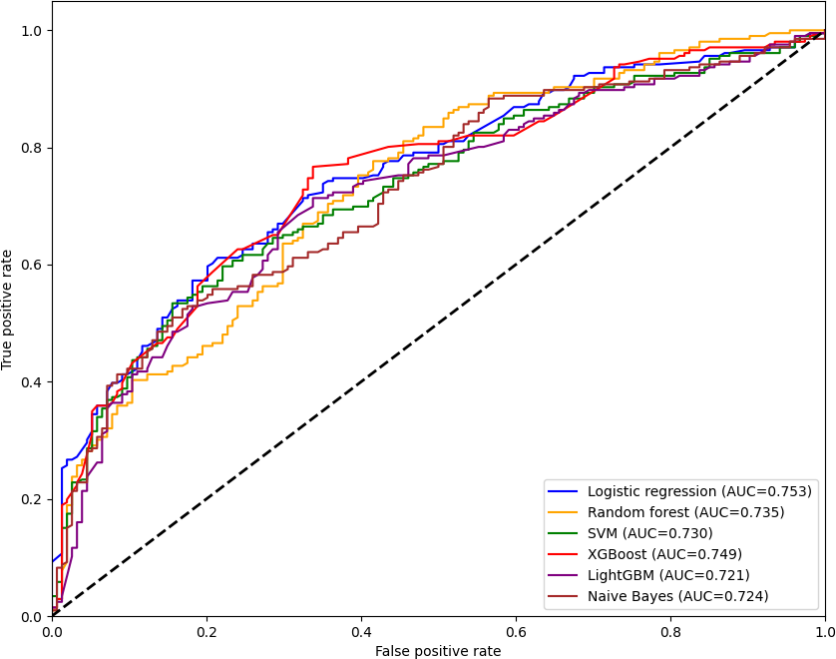
Receiver operating characteristic curves for machine learning models of factors influencing the use of mobile health apps by family caregivers of patients with stroke (combined test set). AUC: area under the curve; LightGBM: light gradient boosting machine; SVM: support vector machine; XGBoost: extreme gradient boosting.

The results of the learning curve for the LR model show that the performance of the training set and the validation set is good and close, indicating that the model fits well (Figure S4 in Multimedia Appendix 1). The final selection of the LR model is 10 features, namely gender, age, marital status, education level, place of residence, whether taking medication, whether the caregiver has a disease, the duration of the patient’s illness, the patient’s self-care ability, and the relationship between the patient and the caregiver. The results of the sensitivity analysis also confirmed the rationality of the feature selection results (Figures S5-S6 in the Multimedia Appendix 1). Figure 2 presents the combined receiver operating characteristic curves of these models on the combined validation set.

### Factors Influencing mHealth App Use Behavior

The ML results show that among the 6 models, RF performed the best (Table S3 in Multimedia Appendix 1). Compared with other models, the RF model had the highest AUROC value (0.773), indicating that this model has the most stable overall ability in classifying categories. RF also has a high accuracy rate and achieves a balance between sensitivity and specificity. The calibration curve and the Brier score also suggest that RF is the best model (Figure S7 in Multimedia Appendix 1). Therefore, after considering all the indicators comprehensively, this study subsequently chose RF for interpretability analysis.

**Figure 3.**
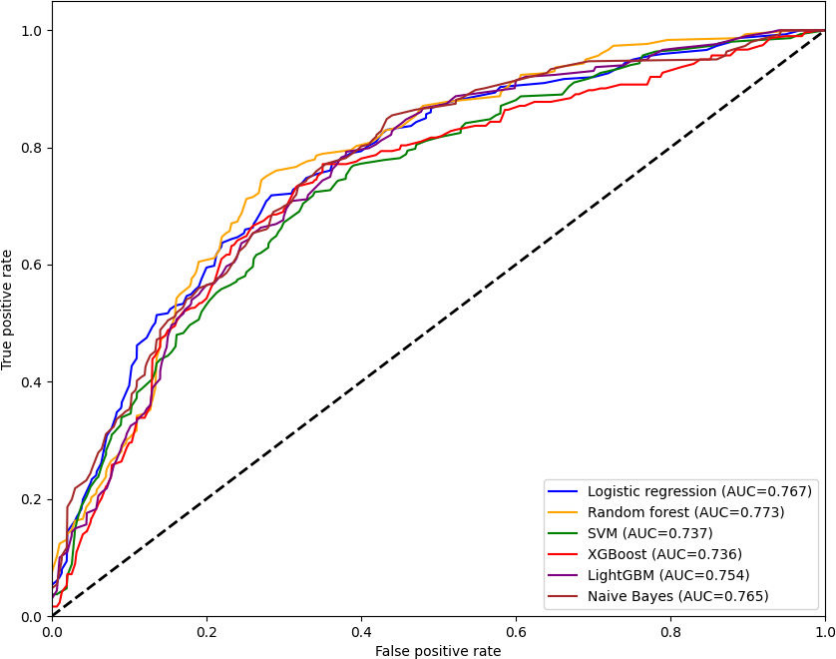
Receiver operating characteristic (ROC) curves for a machine learning model of factors influencing mobile health (mHealth) app use behavior among family caregivers of stroke patients (combined test set). AUC: area under the curve; LightGBM: light gradient boosting machine; SVM: support vector machine; XGBoost: extreme gradient boosting.

The results of the learning curve for the RF model show that the model has gradually converged, the results in the validation set tend to be stable, and there is a small difference from the training set, indicating that the model fits well (Figure S8 in Multimedia Appendix 1). The final selection of the RF model is 8 features, namely income, performance expectation, effort expectation, convenience conditions, hedonic motivation, price value, perceived risk, and habit. The results of the sensitivity analysis also confirmed the rationality of the feature selection results (Figures S9-S10 in the Multimedia Appendix 1). Figure 3 shows the combined receiver operating characteristic curve of these models on the combined test set.

### Interpretability Analysis of Whether to Use mHealth Apps (LR Model)

**Figure 4.**
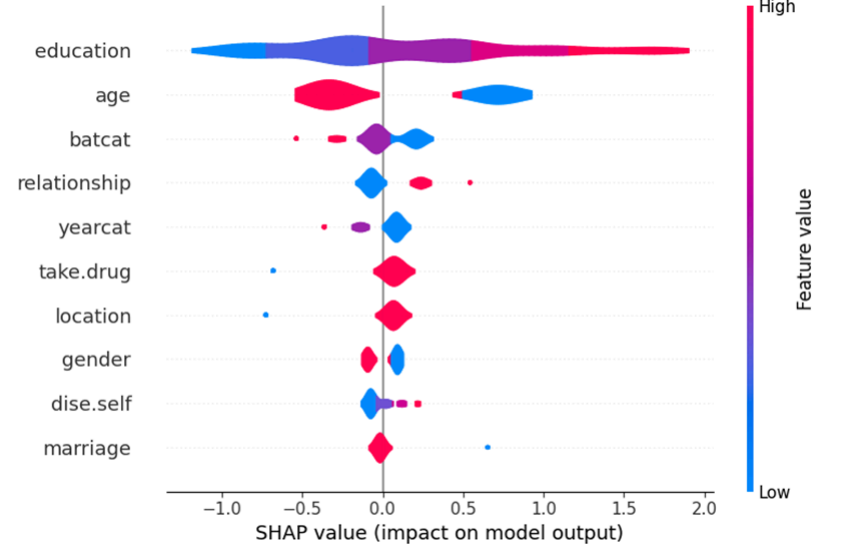
Shapely Additive Explanations (SHAP) summary plots of logistic regression models on whether to use mobile health (mHealth) apps.

We used the SHAP method to interpret the LR model, calculating SHAP values to identify influential features for the overall classification model. The top 5 most influential characteristics were educational level, age, the patient’s self-care ability, the relationship with the cared-for individual, and the duration of illness (Figure S11 in Multimedia Appendix 1). Figure 4 is a SHAP summary plot. The colors in the figure represent the eigenvalue sizes of each participant. Blue represents lower eigenvalues, purple represents medium eigenvalues, and red represents higher eigenvalues. The positive correlation is left blue and right red, while the negative correlation is left red and right blue [31]. The results show that a lower age of the caregiver, a higher educational level of the caregiver, and the caregiver being the patient’s child will have a positive impact on the use of mHealth apps by family caregivers. On the other hand, a lower self-care ability of the patient and a shorter duration of illness will have a negative impact on the use of apps by family caregivers.

### Interpretability Analysis of the Use Behavior of mHealth Apps (RF Model)

**Figure 5.**
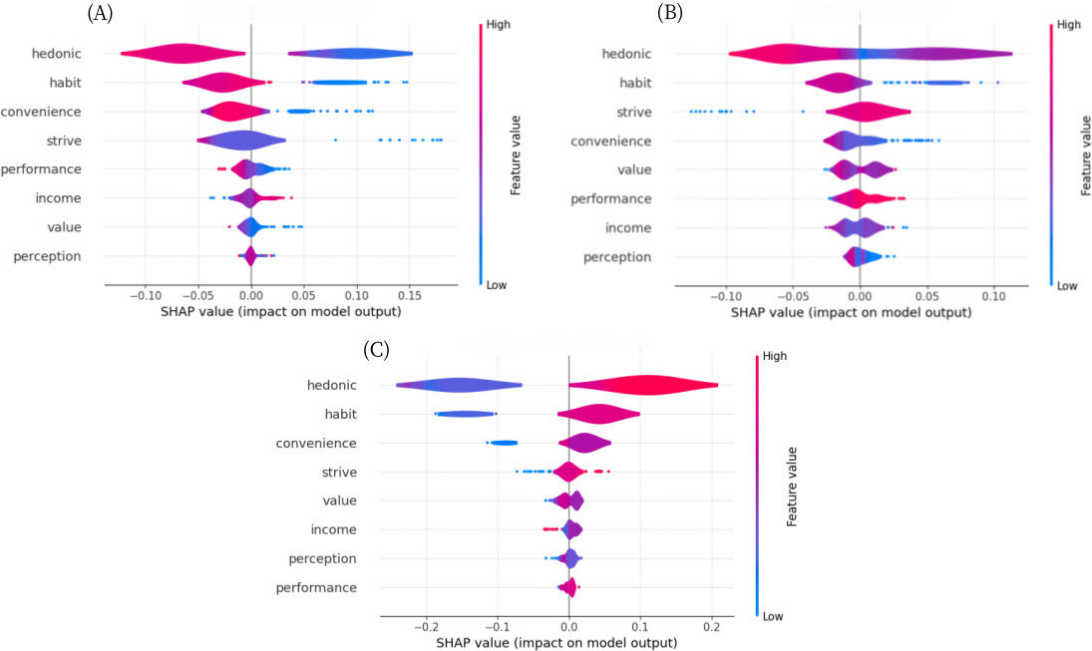
Shapely Additive Explanations (SHAP) summary plots for random forest models of factors influencing mobile health app use behavior. (A) SHAP swarm diagram of cluster 1; (B) SHAP swarm diagram of cluster 2; and (C) SHAP swarm diagram of cluster 3.

We used the SHAP approach to interpret the RF model. The top 5 most influential characteristics were hedonic motivation, habits, convenience conditions, strive expectations, and value (Figure S12 in Multimedia Appendix 1). As the use behaviors were grouped into 3 clusters, summary plots were drawn separately for each cluster to facilitate an understanding of the mechanisms of influence of the different influences within each cluster, as illustrated in Figure 5.

Cluster 3 is the cluster with the most ideal usage behavior among the 3 clusters (ie, the one with the highest use frequency). In order to conduct more targeted research on the influencing factors that promote the use behavior of caregivers and provide precise improvement strategies, this study will take cluster 3 as the basis and combine the research results of cluster 1 to comprehensively determine the 5 most notable influencing factors. The results show that the decline in the caregivers’ enjoyment motivation, use habits, convenience conditions, effort expectations, and performance expectations had a negative impact on the use frequency.

## Discussion

### Overview

The study investigated the status of mHealth app use and factors influencing the use behavior of community-dwelling family caregivers of patients with stroke. We found that the usage rate of mHealth apps among family caregivers of patients with stroke in the community was 57.22%. In terms of whether to use mHealth apps, educational level, age, self-care ability, relationship with the cared-for person, and years of illness were several of the most influential factors. Hedonism, usage habits, convenient conditions, effort expectations, and performance expectations were important influencing factors of use behavior.

Family caregivers provide the core support for the health management of patients with stroke, and they have the responsibility of finding information about stroke care, rehabilitation training, and medication management [32]. An mHealth app can provide them with a source of information to help them better care for patients with stroke [33]. In addition to this, long-term caregiving can lead to emotional and psychological stress for carers [32]. Features including psychological support, stress management, and social connection integrated into mHealth apps can help to improve the psychological state of caregivers and enhance the effectiveness of caregiving. Despite the numerous benefits of mHealth apps, in reality, nearly half of the caregivers have not used any. This indicates that in promoting mHealth apps, we still face many challenges. Therefore, it is crucial to explore the factors that affect the use of mHealth apps.

Lower age and higher literacy levels of caregivers positively influenced the use of mHealth apps among family caregivers. This may occur because younger, more literate caregivers tend to show greater acceptance and familiarity with mHealth apps [34]. This positive attitude toward technology and knowledge of emerging health management tools can help promote their use of mHealth apps [35,36]. Not only that, but this group also tends to be children of patients. Previous research has found that care-dependent older adults and their adult children are important determinants of predicting and supporting the use of smart technology [37]. Our study validates this idea, which may be because younger-generation offspring are more tolerant and adaptive to new technologies and devices compared to the patient’s partner or parents. In contrast, partners and parents who are relatively older and less educated are affected by cognitive decline and the deterioration of their learning and memory abilities, and hold fearful and skeptical attitudes toward new technologies such as mHealth apps [38].

A shorter duration of illness and poorer self-care ability can have a negative impact on the use of mHealth apps by caregivers. Previous studies have found that a lack of understanding of mHealth apps and insufficient time are significant obstacles to their use [39]. The results of this study support this view. Patients with shorter diagnosis periods are in the adaptation stage, and caregivers may focus more on basic treatment rather than preventive management, thus having a lower willingness to use mHealth apps. Poorer self-care can negatively affect caregiver use of mHealth apps. Patients with poor self-care skills require a significant investment of time and energy from their caregivers. Caregivers may be busy all day long helping patients with daily living activities, such as washing, feeding, and turning, and they also need to keep an eye on changes in the patient’s condition. This leaves them little free time to learn and use mHealth apps.

The study results reveal the existence of the digital divide. These vulnerable groups in the digital society cannot benefit from technological progress. If digital technology–based intervention measures fail to fully consider the obstacles faced by vulnerable groups, such as technological fears and lack of knowledge, when being designed and promoted, they may unintentionally exacerbate health inequalities [40-42]. This may lead to the groups that are most in need of support (such as low-income, high-burden, and older caregivers) being excluded from the benefits brought by digital health, resulting in “digital exclusion.” To alleviate this phenomenon, in addition to designing more intuitive and user-friendly apps with lower costs, it is also necessary to explore hybrid support models (such as combining apps with the guidance of community workers or nurses), provide digital training, and develop alternative solutions with low technical barriers (such as SMS text messaging services and voice interaction), so that they can obtain digital technology services even in resource-poor environments.

Hedonic motivation emphasizes the pleasurable experience and satisfaction gained through the use of mHealth apps [43]. When carers experience pleasure, perceived caregiving stress, and reduced psychological burden through the use of mHealth apps, they tend to use these tools more frequently, thus contributing to increased frequency of use. Habit formation significantly influences decision-making processes and behavioral patterns [44]. Once a habit is formed, individuals tend to persist in that pattern of behavior because changing established habits requires more cognitive effort and willpower [45]. If caregivers have developed the habit of seeking help and accessing health information on mHealth apps whenever they encounter health problems related to the patient and themselves, this behavior becomes part of their lives. Therefore, when promoting mHealth apps, it is crucial to value and promote the formation of positive habits of use, which can help to transform the use of mHealth apps and incorporate them into the routines of daily life [46].

Convenient conditions refer to the available resources and support levels for using mHealth apps [47]. Factors such as the lack of equipment, insufficient training, and social support can all increase the barriers to using mHealth apps [48,49]. Therefore, it is suggested that when promoting mHealth apps, we should take into account external factors such as increasing facilities to improve the use behavior. Performance expectations, on the other hand, focus on carers’ assessment of the expected benefits from using mHealth apps. If carers believe that using mHealth apps will significantly help them reduce the burden of caring for patients, such as receiving personalized rehabilitation advice, facilitating communication with doctors, and obtaining psychological relief and social support, their use behavior would improve in all 3 areas. Effort expectancy refers to the degree of cognitive and behavioral effort an individual perceives is required to use a particular technology [50]. If carers recognize their abilities and believe they can master and effectively use mHealth apps, they are likely to have a higher willingness to use the tools and be more motivated to use them [51].

In addition, considerations related to data privacy should also be taken seriously. mHealth apps usually need to collect sensitive medical and personal data. If these data are leaked or improperly used, it not only infringes upon personal privacy but may even lead to discrimination or psychological harm [52,53]. Therefore, developers and service providers must attach importance to and provide privacy protection mechanisms (such as anonymization processing, clear data use, and sharing agreements) to ensure that users can conveniently control their data access and deletion permissions.

### Limitations

This study has several limitations that should be considered when interpreting the results. First, to maximize participant recruitment, the study used convenience sampling, which may limit sample representativeness. To enhance generalizability and verify the broad applicability of the conclusions, it is recommended that future studies adopt a more diverse and comprehensive sampling strategy to ensure that the sample covers individuals with different characteristics and backgrounds. Second, this study just focused on the overall use and the influencing factors of mHealth apps by family carers of patients with stroke and did not conduct in-depth analyses for specific types of apps. Future research could consider exploring the specific use and influencing factors of family carers of patients with stroke on various types of apps to better meet the specific needs of users. It is worth noting that although some stable analytical results have been achieved so far, the sample of this study is limited to the northern region of China. In the future, data from multicenter large samples will be further collected to conduct more in-depth and extensive research.

Although this study innovatively adopted ML algorithms for data analysis, it is necessary to acknowledge the inherent limitations of ML methods. First, despite the use of the nested cross-validation method, the risk of overfitting still cannot be completely avoided, especially in small sample datasets. Second, although models trained based on data from a single region exhibit internal stability, their external generalization ability may be limited by regional factors, thereby affecting the model’s applicability. Furthermore, ensemble algorithms such as RFs, while providing highly accurate predictions, still pose challenges to their interpretability due to their “black box” decision-making characteristics. Although we incorporated SHAP analysis, the complexity of this post hoc explanation is still higher than that of models that are inherently transparent. Therefore, it is suggested that future research should conduct multicenter studies and expand the scale of the dataset to support the research of process-explainable large-scale ML methods, such as the Kolmogorov-Arnold networks.

### Conclusions

This study focused on the use of mHealth apps by family caregivers of patients with stroke in the community and the factors influencing their use behavior. The research found that caregivers with advanced age, low education, shorter illness durations, and heavy care burden are vulnerable groups in digital exclusion, and explored the influencing factors of use behavior, providing a theoretical basis for further optimizing mHealth apps. The results suggest that community workers should take digitally vulnerable groups as the key support targets, actively recommend high-quality mHealth apps to them, emphasize the core value of the apps, and provide practical introductory training to help caregivers overcome initial technical fears and operational difficulties. Software developers should focus on the age-friendly design of software and carry out cost control. Under the premise of ensuring professionalism, feedback mechanisms and interactive elements added should be to enhance the pleasure and satisfaction of use and promote the formation of habits. Meanwhile, importance is attached to data privacy and security, enabling mHealth apps to truly benefit patients with stroke and their family caregivers.

## Supplementary material

10.2196/73903 Multimedia Appendix 1 Supplementary materials regarding the data analysis, figures, and tables involved in the research.

## Abbreviations

AUROC: area under the receiver operating characteristic
EPV: events per variable
LR: logistic regression
mHealth: mobile health
ML: machine learning
RF: random forest
SHAP: Shapley Additive Explanations
