# Supplementary material for: Interpretable Machine Learning Models for Analyzing Determinants Affecting the Use of mHealth Apps Among Family Caregivers of Patients With Stroke in Chinese Communities: Cross-Sectional Survey Study
Source: JMIR Mhealth Uhealth. 2025 Nov 24;13:e73903. doi: 10.2196/73903 (PMC12643396; doi:10.2196/73903)
Supplement: Multimedia Appendix 1 [file mhealth-v13-e73903-s001.docx]

**Supplementary methodology, figures and tables.**

**Questionnaire survey**

The performance expectations, effort expectations, social impact, and perceived risk were measured using the questionnaire designed by scholar Yang Qiuxia [1]. Among them, there were 4 items for performance expectations, 3 items for effort expectations, 3 items for social impact, and 5 items for perceived risk. The Likert 5-point scale scoring method was adopted. The higher the score, the greater the recognition of the corresponding factors. The Cronbach's α coefficient for performance expectations was 0.861, for effort expectations was 0.924, for social impact was 0.795, and for perceived risk was 0.772.

The questionnaire designed by scholar Liu Ye was used to assess facilitating conditions and price value [2]. Facilitating Conditions consisted of four items, while Price Value included three items. A five-point Likert scale was adopted for scoring, with higher scores indicating better facilitating conditions and higher price value. The Cronbach's α for Facilitating Conditions was 0.826, and for Price Value, it was 0.803.

Hedonic motivation was assessed using the questionnaire designed by scholar Zhou Yuan [3]. This questionnaire consists of 3 items and employs a five-point Likert scale for scoring. Higher scores indicate a higher level of hedonic motivation. The Cronbach's α for this questionnaire was 0.827.

Habit was assessed using the questionnaire designed by scholar Yin Shuhui [4]. The questionnaire consists of 3 items, employing a five-point Likert scale for scoring. Higher scores indicate a greater degree of habit, and the Cronbach's α for this questionnaire was 0.884.

The questionnaire designed by scholar Peng Qian was used to measure behavioral intention to use [5]. It consists of 4 items and employs a five-point Likert scale for scoring. Higher scores indicate a stronger behavioral intention to use, and the questionnaire demonstrated a Cronbach's α of 0.911.

**The selection of k-modes clustering algorithm**

The k-modes clustering method is an extension and expansion of the k-means clustering algorithm, and it is a clustering analysis specifically designed for classification attribute datasets. The difference between this algorithm and the k-means clustering lies in that the cluster centers are no longer the mean of the belonging points, but the mode of the belonging points. In terms of calculating distances, it no longer uses Euclidean distance, but compares the differences in each attribute of the point and the attribute of the cluster center to determine the distance from the point to the cluster center. The basic steps of this algorithm are to randomly determine k cluster centers, then compare the distances between the remaining samples and each cluster center (the length of the distance here is the number of different attributes between the sample point and the cluster center), find the cluster closest to them for clustering, and recalculate the center of each cluster. Repeat the iteration until the sum of the distances between the samples in each cluster and their respective cluster centers no longer decreases, thereby obtaining the best clustering result. This algorithm is simple in principle and has high computational efficiency. Compared with the k-means clustering, it is more suitable for the classification data in this study. Not only that, k-modes clustering also belongs to unsupervised machine learning. Compared with supervised learning, unsupervised learning does not use manually set data labels. It can identify the potential correlations and structures among high-dimensional data, thereby dividing the data set into relevant clusters [1]. This can provide structured information for subsequent machine learning and help the model be better constructed [2, 3]. Therefore, in this study, the k-modes clustering algorithm is selected and the research objects of the mHealth App will be classified.

**Elbow method**

In the traditional K-modes algorithm, the determination of the number of clusters often relies on manual judgment. This subjectivity greatly affects the accuracy and reliability of clustering. The elbow method can avoid this problem. The principle of this method is that as the number of clusters k increases, the division of the data will become more detailed, and the tightness of each cluster will gradually increase. Therefore, the Sum of Squared Errors (SSE) will gradually decrease. When the k value is less than the actual number of clusters in the data, increasing the k value will significantly enhance the density of the clusters, resulting in a significant reduction in SSE. However, once the k value reaches the true number of clusters of the data, the improvement in compactness brought about by further increasing the k value will decrease sharply. Therefore, the reduction amplitude of SSE will suddenly become smaller, and then tend to stabilize as the k value increases, forming a shape similar to an elbow. The k value corresponding to this elbow is the potential number of clusters of the data.

**The principles and selection of machine learning models**

LR is one of the most widely used machine learning classification algorithms and is often used to solve binary classification problems. RF is a classifier containing multiple decision trees, and its principle is to summarize and process the randomly generated multiple decision trees. This algorithm has a good tolerance for outliers and noise and has better prediction and classification performance compared with decision trees [4]. SVM finds the optimal segmentation hyperplane by maximizing the margin between support vectors, so the generalization error rate of this algorithm is low [5]. XGBoost is a type of Boosting algorithm. It builds a set of decision trees by iteratively focusing on subsets of training data that are more difficult to predict [6]. LightBGM is also a type of Boosting algorithm. However, it adopts the Leaf growth strategy of Leaf-wise with depth limitations [7], enabling it to control the model complexity in small samples and avoid overfitting. NB is a generative model that simplifies the learning process through the assumption of independence among features. This algorithm is relatively simple and has a low theoretical misjudgment rate at the same time. It is still effective when the data is small [8]. These machine learning algorithms are widely used in various complex data analysis tasks due to their excellent generalization and discrimination capabilities in processing data, and remain effective on small sample datasets [9, 10]. Therefore, the above six algorithms were selected for analysis in this study.

**Introduction to SHAP Algorithm**

SHAP is a game theory-based method that can provide consistent and accurate feature contribution calculations for the predictions of any machine learning model by using Shapley values [11]. To calculate the Shapley value of a feature, the difference between the predicted value generated when the feature exists and the predicted value generated when the feature does not exist is first calculated. This difference is called the marginal contribution value of the feature to the current prediction. SHAP, on the other hand, constructs an additive interpretation model. It calculates the marginal contribution value of the feature in all combinations that include or exclude it and obtains the average value, which is the Shapley value.

**The range of parameter adjustments for the model and the selected optimal parameters**

1) Whether to use the mHealth App

Logistic Regression:

threshold: ['median', 'mean']

C: [0.1, 1, 10]

penalty: ['l2']

Random Forest:

threshold: ['median', 'mean']

n_estimators: [50, 100]

max_depth: [None, 10]

min_samples_split: [2, 5]

SVM:

C: [0.1, 1]

kernel: ['linear', 'rbf']

n_features_to_select: [10, 15]

XGBoost:

n_estimators: [50, 100]

max_depth: [3, 6]

learning_rate: [0.01, 0.1]

max_depth: [3]

LightGBM:

n_estimators: [50, 100]

max_depth: [3, 6]

learning_rate: [0.01, 0.1]

num_leaves: [15]

Naive Bayes:

k: [8, 10, 12]

n_components: [0.85, 0.9, 0.95]

The selected optimal model is the LR model, and the best parameters are 'clf__C': 0.1, 'clf__penalty': 'l2', 'selector__threshold': 'median'.

2) Use behavior of mHealth App

Logistic Regression:

threshold: [1.25*median]

C: [0.1, 1, 10]

class_weight': [None, class_weights]

Random Forest:

threshold: [1.5*median]

n_estimators: [50]

max_depth: [3, 5]

class_weight': [None, class_weights]

SVM:

C: [0.01, 0.1, 1]

class_weight': [None, class_weights]

XGBoost:

threshold: [1.5*median]

n_estimators: [50]

max_depth: [3, 5]

class_weight': [None, class_weights]

LightGBM:

threshold: [1.5*median]

n_estimators: [50]

max_depth: [3, 5]

class_weight': [class_weights]

Naive Bayes:

k: [8, 10]

n_components: [0.95]

The selected optimal model is the RF model, and the best parameters are 'clf__class_weight': None, 'clf__max_depth': 3, 'clf__n_estimators': 50

**Table S1 The feature selection method of machine learning models.**

| Model | Feature Selection |
| --- | --- |
| LR | L1 regularization |
| RF | Random Forest Feature Importance |
| SVM | Recursive feature elimination |
| XGBoost | Feature importance selection based on gain |
| LightGBM | Number of divisions |
| NB | Analysis of Variance filtering and PCA dimensionality reduction |

**Table S2 Performance of a machine learning model of factors influencing whether family caregivers of stroke patients use mHealth APP**

| Model | Accuracy | AUC | Sensitivity | Specificity |
| --- | --- | --- | --- | --- |
| LR | 0.694 (0.647-0.742) | 0.753 (0.698-0.802) | 0.748 (0.688-0.806) | 0.623 (0.547-0.698) |
| RF | 0.683 (0.636-0.736) | 0.735 (0.681-0.786) | 0.752 (0.693-0.812) | 0.591 (0.510-0.665) |
| SVM | 0.647 (0.600-0.692) | 0.730 (0.673-0.780) | 0.782 (0.721-0.833) | 0.468 (0.396-0.545) |
| XGBoost | 0.706 (0.656-0.753) | 0.749 (0.696-0.797) | 0.772 (0.716-0.829) | 0.617 (0.535-0.693) |
| LightGBM | 0.667 (0.619-0.717) | 0.721 (0.663-0.772) | 0.762 (0.701-0.817) | 0.539 (0.461-0.613) |
| NB | 0.633 (0.586-0.683) | 0.724 (0.672-0.777) | 0.636 (0.572-0.700) | 0.630 (0.548-0.702) |

**Table S3 Performance of a machine learning model of mHealth App usage behavior for family caregivers of stroke patients.**

| Model | Accuracy | AUC | Sensitivity | Specificity |
| --- | --- | --- | --- | --- |
| LR | 0.578 (0.510-0.646) | 0.767 (0.718-0.819) | 0.432 (0.385-0.484) | 0.727 (0.703-0.753) |
| RF | 0.602 (0.534-0.665) | 0.773 (0.725-0.818) | 0.476 (0.420-0.533) | 0.769 (0.738-0.797) |
| SVM | 0.515 (0.446-0.583) | 0.737 (0.683-0.788) | 0.333 (0.333-0.333) | 0.667 (0.667-0.667) |
| XGBoost | 0.607 (0.544-0.675) | 0.736 (0.685-0.793) | 0.535 (0.471-0.602) | 0.797 (0.764-0.829) |
| LightGBM | 0.597 (0.529-0.660) | 0.754 (0.702-0.802) | 0.560 (0.488-0.626) | 0.791 (0.756-0.826) |
| NB | 0.617 (0.553-0.680) | 0.765 (0.716-0.817) | 0.561 (0.491-0.631) | 0.795 (0.761-0.828) |


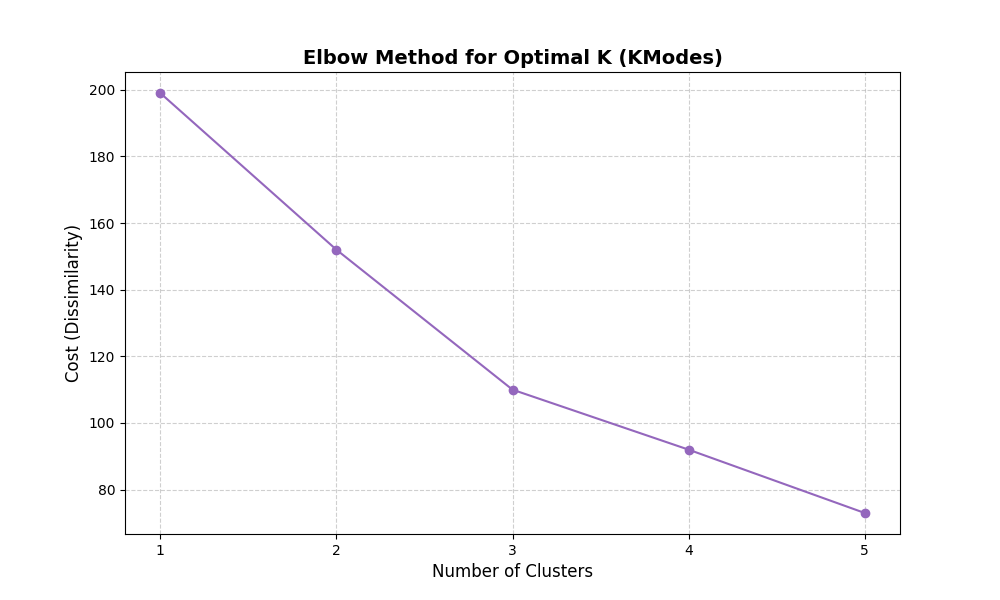


Figure S1 Scree plot based on the number of clusters.


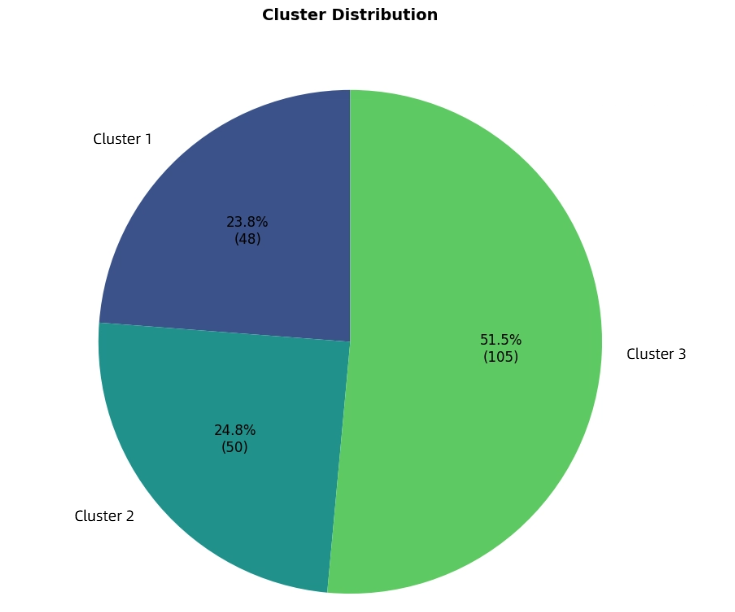


Figure S2 The distribution of the number of people in different clusters. Among them, cluster 3 represents the group with the best performance in terms of usage behavior.


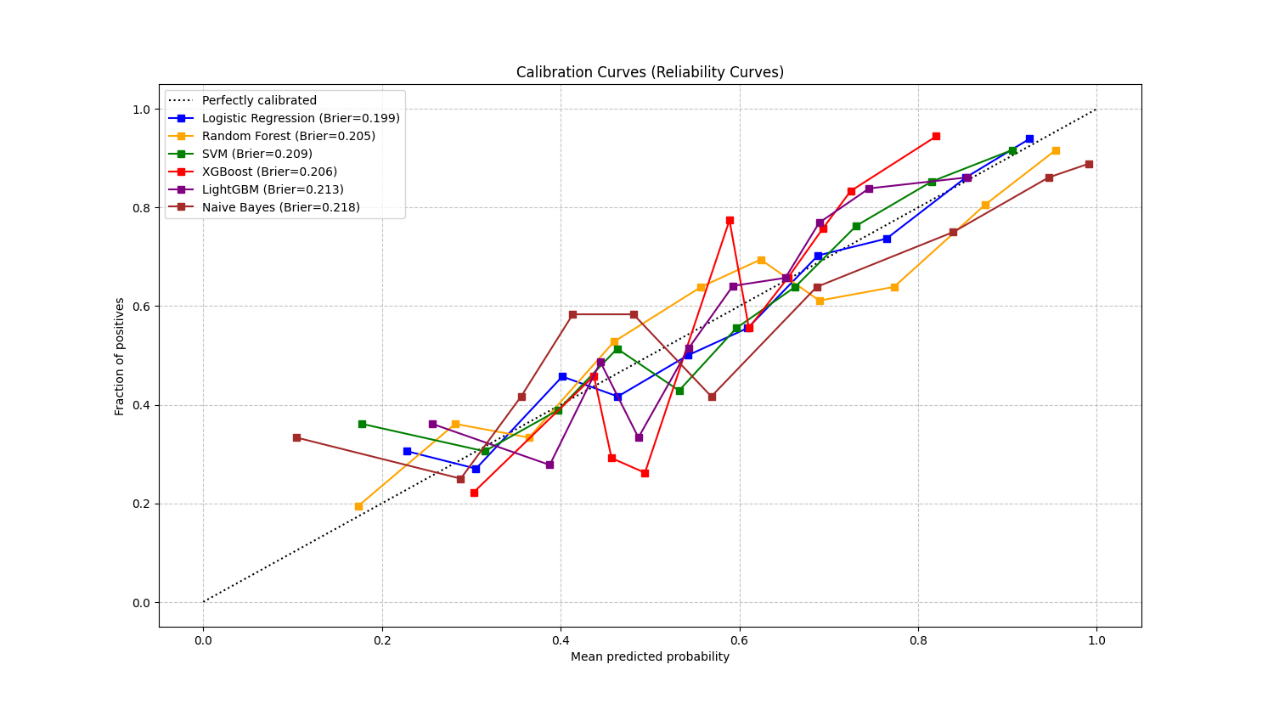


Figure S3 Calibration curve and Brier score of LR models on the use of mHealth App or not. Among them, the model that is closer to the ideal line (i.e., the diagonal focal dotted line) has better calibration. The smaller the Brier value is, the better the calibration of the model is. From the figure, it can be seen that the LR model has the best calibration.


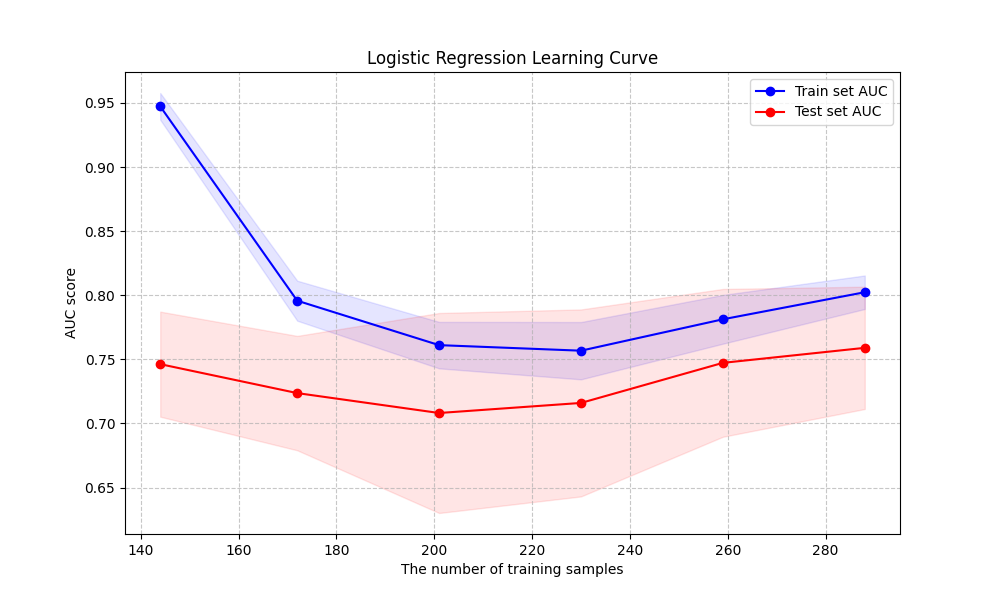


Figure S4 Learning curve of LR models on the use of mHealth App or not. The shaded area in the figure represents the standard deviation. The results show that as the number of training samples increases, the AUC value of the test set gradually stabilizes, and the gap with the training set gradually narrows.


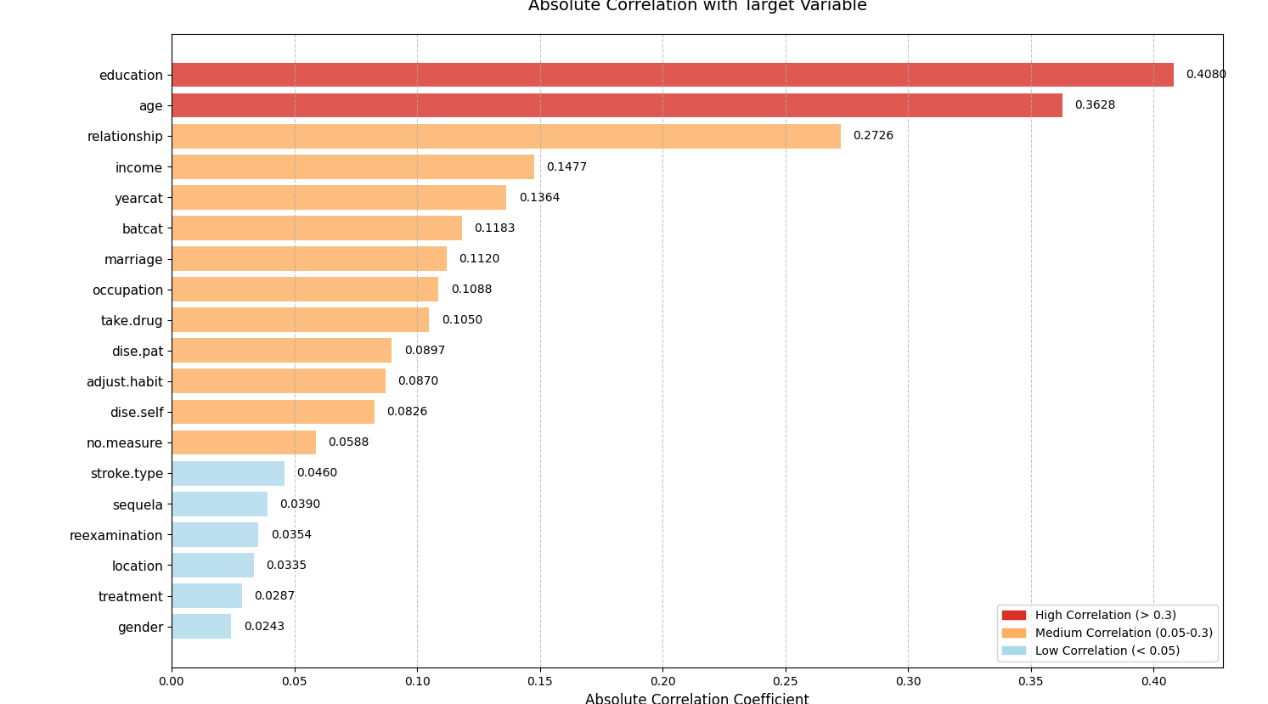


Figure S5 Absolute correlation between the features and target variable on the use of mHealth App or not.


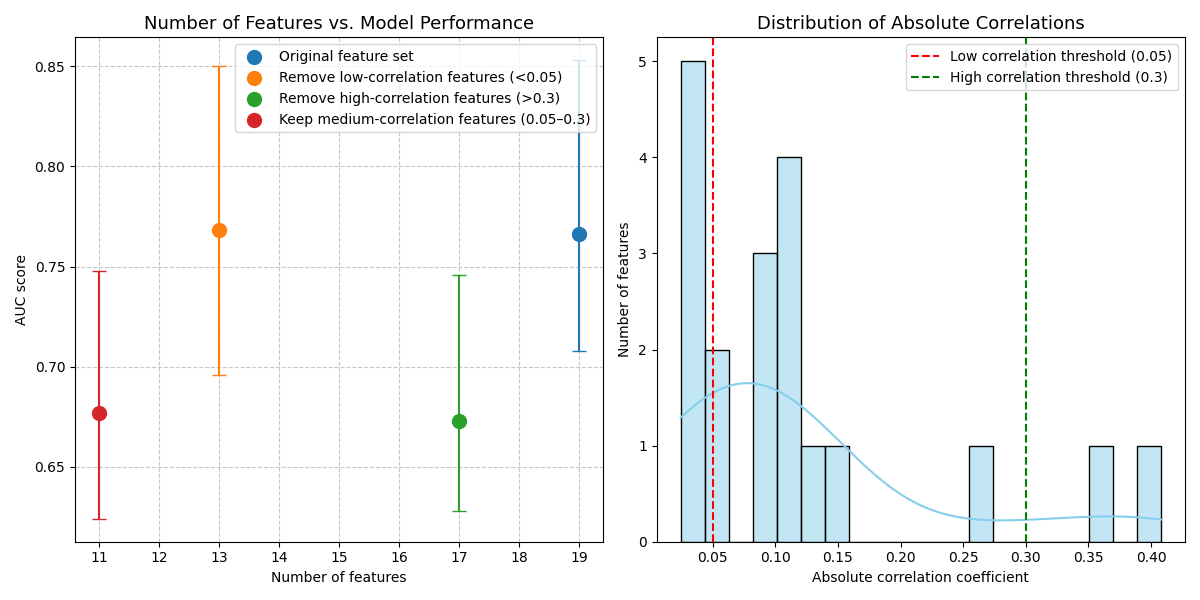


Figure S6 The relationship between the number of features and model performance (model on the use of mHealth App or not). The results show that removing highly correlated features has a significant impact on the model performance, resulting in a decline in performance. Removing lowly correlated features has a relatively minor impact on the model performance. Models that only retain moderately correlated features have relatively stable performance, but are slightly lower than those with the original feature set. Among the 10 selected features of the model ("gender", "age", "marital status", "education level", "location", "drug use behavior", "self-health condition", "year category", "monthly category", "relationship"), all highly correlated variables, most moderately correlated variables, and all low-correlation variables have been excluded. This indicates that the selected features by the model are reasonable.


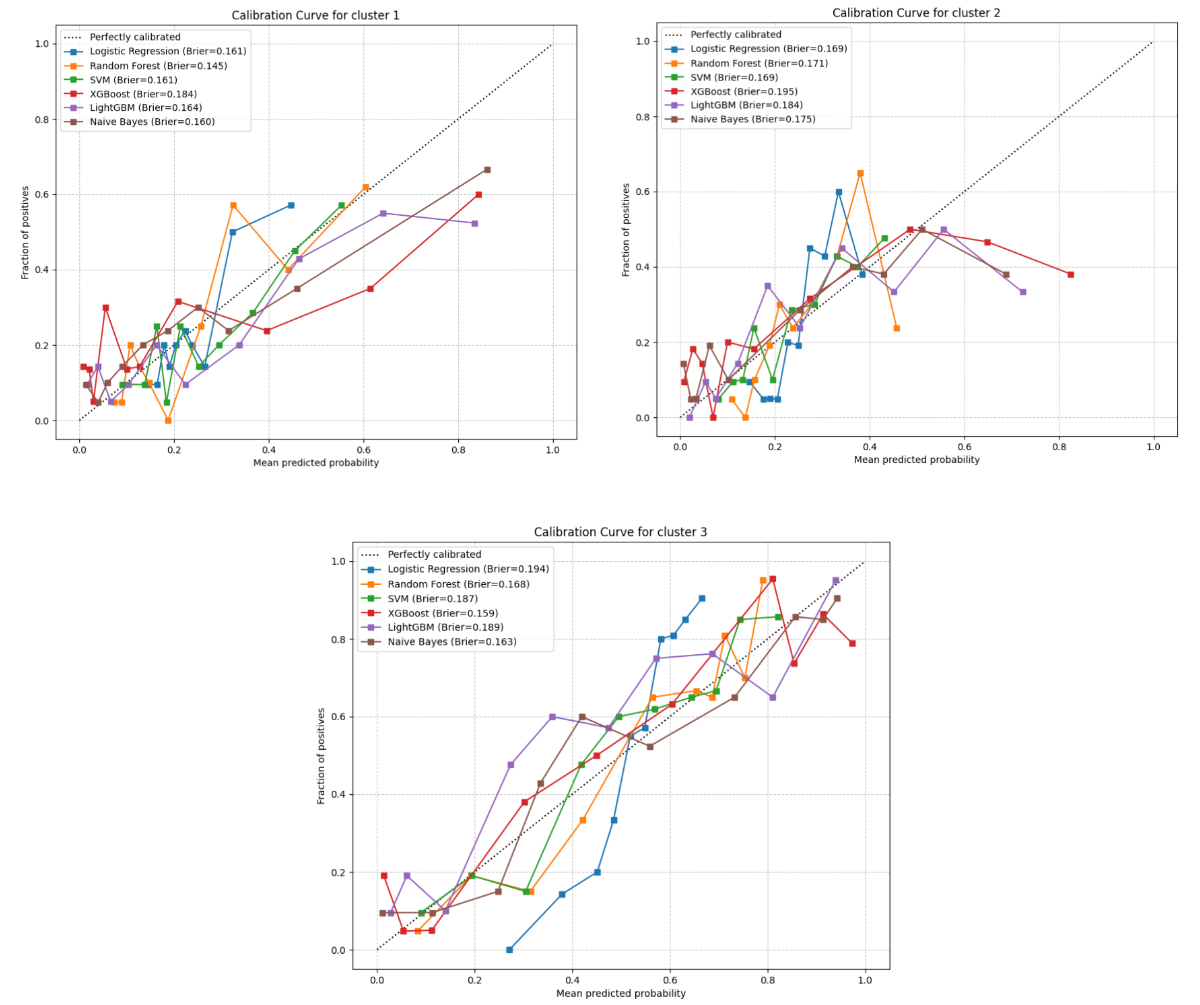


Figure S7 Calibration curve and Brier score of RF models on the use behavior of mHealth App. Random Forest demonstrated relatively stable performance across all clusters, with the best calibration effect, particularly approaching the ideal line in the high predicted probability range.


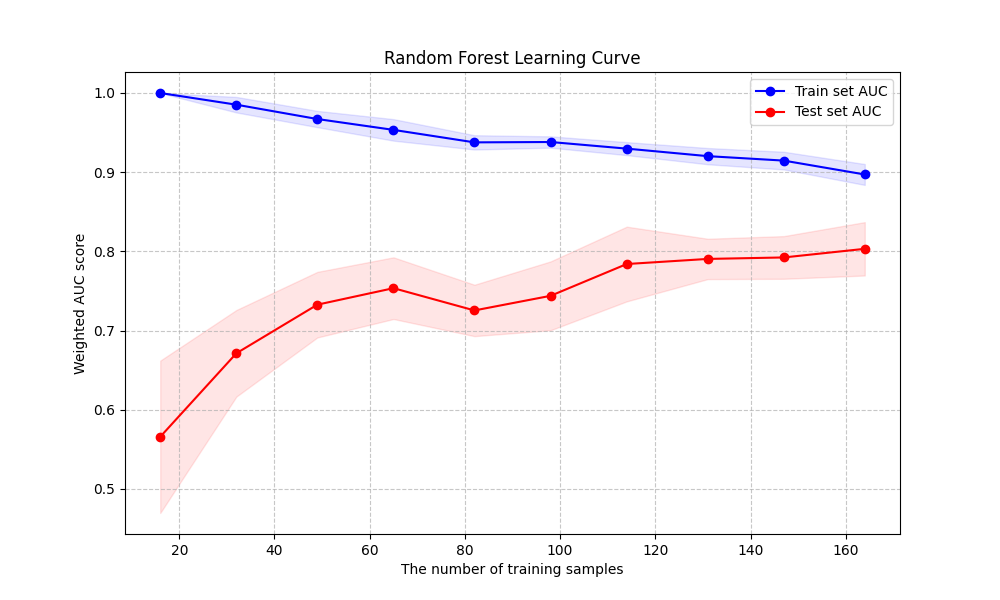


Figure S8 Learning curve of RF models on the use behavior of mHealth App. The shaded area in the figure represents the standard deviation. The result shows that as the sample size increases, the AUC value of the validation set gradually rises to a plateau, and the difference between the validation set and the training set gradually narrows.
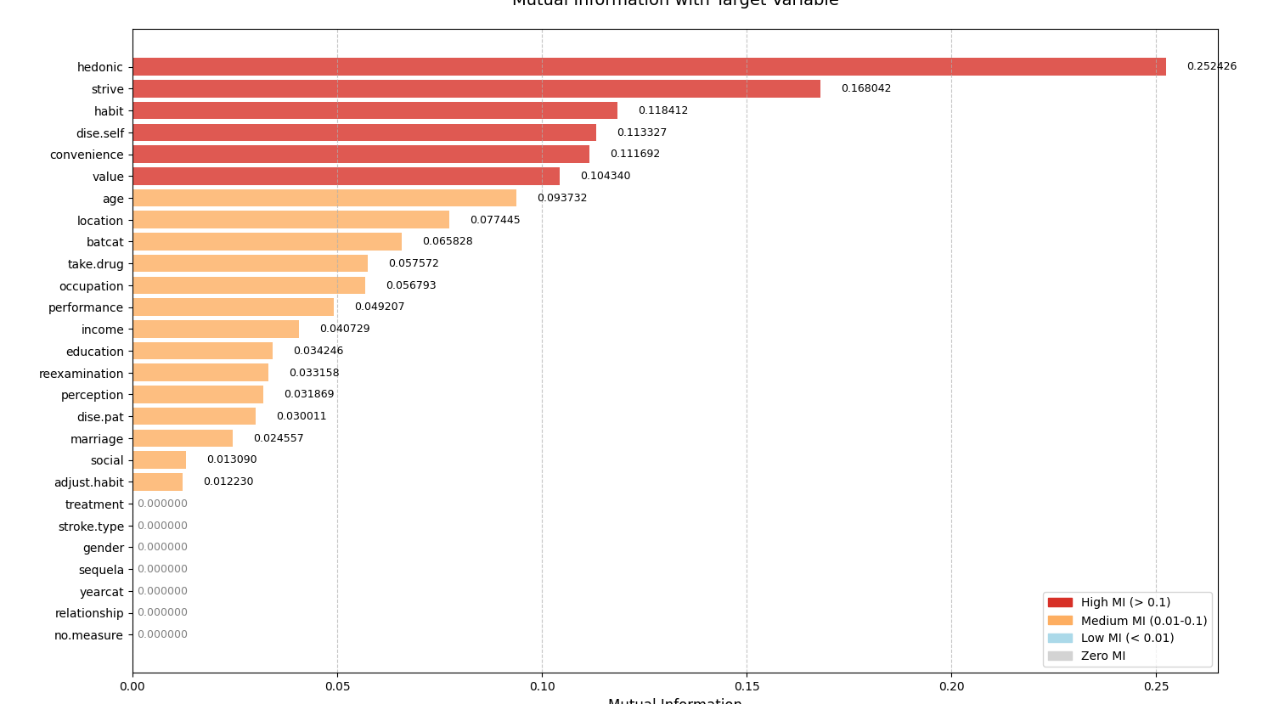


Figure S9 Mutual information between the features and the target variable on the use behavior of mHealth App.


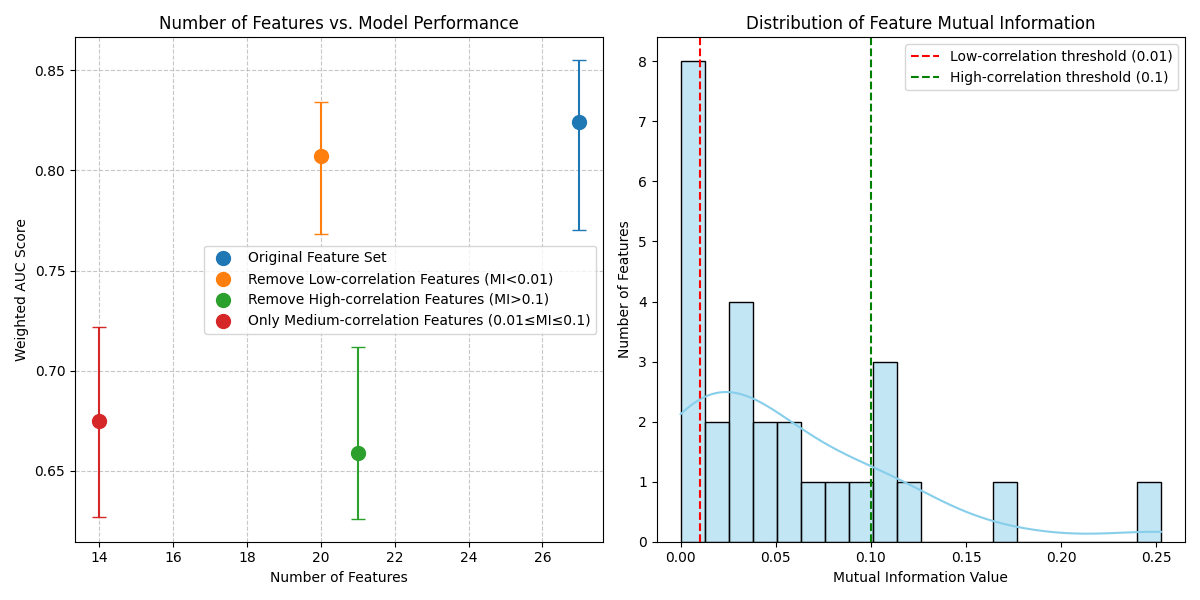


Figure S10 The relationship between the number of features and model performance (model on the use behavior of mHealth App). The results show that removing highly correlated features has a significant impact on the model performance, resulting in a substantial decline in performance. Removing lowly correlated features has a relatively minor effect on the model performance. Models that only retain moderately correlated features have relatively stable performance, but are slightly lower than those with the original feature set. After feature selection in the RF model, 8 variables including 'income', 'performance', 'strive', 'convenience', 'hedonic', 'value', 'perception', and 'habit' were included, covering most of the variables with high mutual information values.


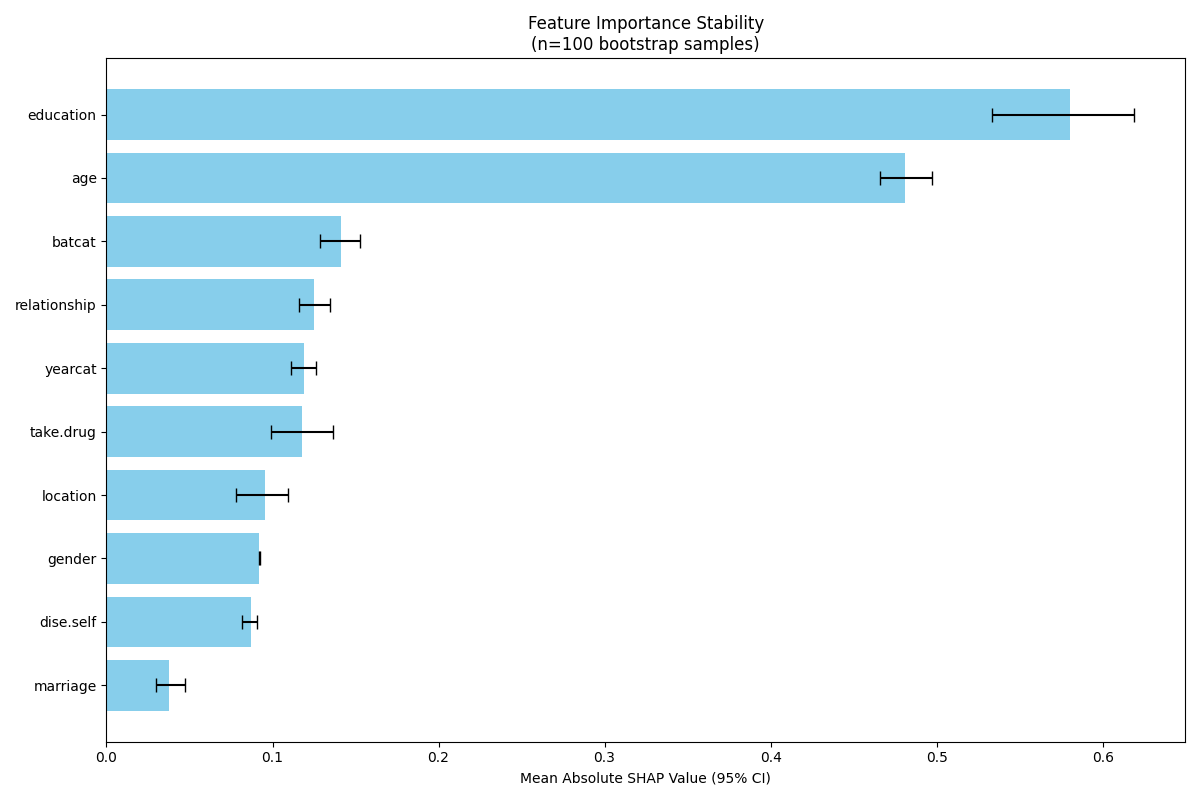


Figure S11 Characteristic importance ranking of LR models on the use of mHealth App or not. Among them, each characteristic is ranked in descending order of importance. The top five most influential characteristics are educational level, age, the patient's self-care ability, the relationship with the cared-for individual, and the duration of illness.


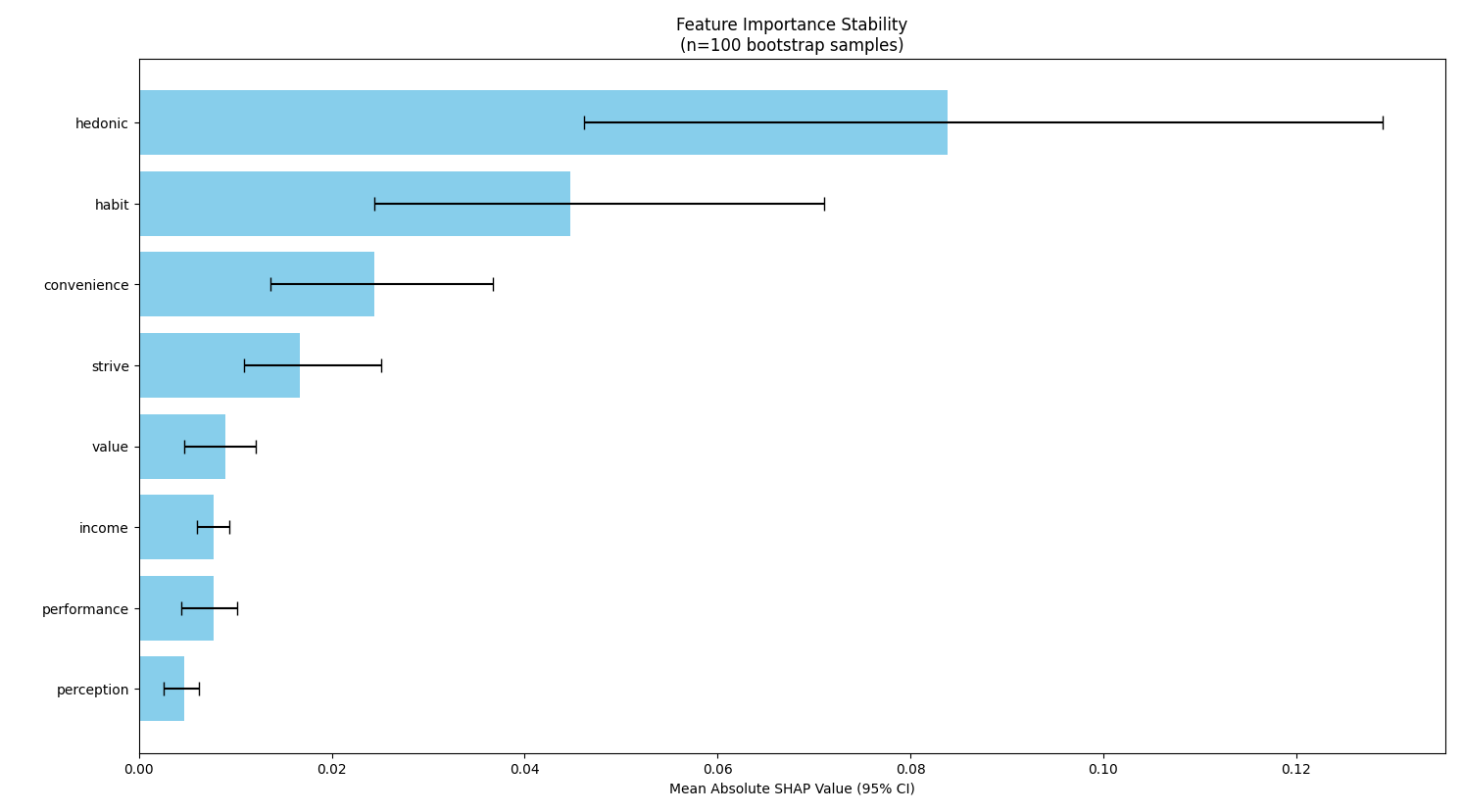


Figure S12 Feature Importance ranking of RF models of factors influencing mHealth App usage behavior. Among them, each feature is arranged in descending order of total importance. The top five most influential features are hedonic motivation, habits, convenience conditions, strive expectations and value.

1. Qiuxia Y. Study on influencing factors of elderly'acceptance of health management systembased on UTAUT model. [Master's degree]: Gansu University Of Chinese Medicine; 2021.

2. Ye L. Research on Influencing Factors of User’s Intention of AR Commercial Application Based-on UTAUT2 Model [Master's degree]: Jinan University; 2021.

3. Yuan Z. Research on Influencing Factors of Users' Behavioral Intentionto Use Paying Courses in Mobile Fitness APP Based on UTAUT2 Model [Master's degree]: Shanghai University of Sport; 2021.

4. Shuhui Y. Investigation on the status and willingness ofelderly patients with cardiovascular disease touse intelligent emergency alert devices [Master's degree]: Hunan Normal University; 2021.

5. Qian P. Research on influencing factors of using and accepting online health community based on UTAUT Model [Master's degree]: Jinan University; 2020.

6. Eckhardt CM, Madjarova SJ, Williams RJ, Ollivier M, Karlsson J, Pareek A, et al. Unsupervised machine learning methods and emerging applications in healthcare. Knee Surg Sports Traumatol Arthrosc. 2023 Feb;31(2):376-81. PMID: 36378293. doi: 10.1007/s00167-022-07233-7.

7. Yang L, Huang B, Guo S, Lin Y, Zhao T. A Small-Sample Text Classification Model Based on Pseudo-Label Fusion Clustering Algorithm. Applied Sciences. 2023;13(8):4716. PMID: doi:10.3390/app13084716.

8. Muhammad Noor Mathivanan N, Md.Ghani N, Mohd Janor R. Improving Classification Accuracy Using Clustering Technique. Bulletin of Electrical Engineering and Informatics. 2018 09/01;7:465-70. doi: 10.11591/eei.v7i3.1272.

9. Breiman L. Random Forests. Machine Learning. 2001 2001/10/01;45(1):5-32. doi: 10.1023/A:1010933404324.

10. Cortes C, Vapnik V. Support-vector networks. Machine Learning. 1995 1995/09/01;20(3):273-97. doi: 10.1007/BF00994018.

11. Li W, Peng Y, Peng K. Diabetes prediction model based on GA-XGBoost and stacking ensemble algorithm. PLoS One. 2024;19(9):e0311222. PMID: 39348356. doi: 10.1371/journal.pone.0311222.

12. Makhmutova M, Kainkaryam R, Ferreira M, Min J, Jaggi M, Clay I. Predicting Changes in Depression Severity Using the PSYCHE-D (Prediction of Severity Change-Depression) Model Involving Person-Generated Health Data: Longitudinal Case-Control Observational Study. JMIR Mhealth Uhealth. 2022 Mar 25;10(3):e34148. PMID: 35333186. doi: 10.2196/34148.

13. Maheswari S, Pitchai R. Heart Disease Prediction System Using Decision Tree and Naive Bayes Algorithm. Curr Med Imaging Rev. 2019;15(8):712-7. PMID: 32008540. doi: 10.2174/1573405614666180322141259.

14. Li X, Ding F, Zhang L, Zhao S, Hu Z, Ma Z, et al. Interpretable machine learning method to predict the risk of pre-diabetes using a national-wide cross-sectional data: evidence from CHNS. BMC Public Health. 2025 Mar 26;25(1):1145. PMID: 40140819. doi: 10.1186/s12889-025-22419-7.

15. Campagner A, Berjano P, Lamartina C, Langella F, Lombardi G, Cabitza F. Assessment and prediction of spine surgery invasiveness with machine learning techniques. Comput Biol Med. 2020 Jun;121:103796. PMID: 32568677. doi: 10.1016/j.compbiomed.2020.103796.

16. Lundberg SM, Lee S-I, editors. A Unified Approach to Interpreting Model Predictions. Neural Information Processing Systems; 2017.
